# The molecular mechanisms of remodeling in asthma, COPD and IPF with a special emphasis on the complex role of Wnt5A

**DOI:** 10.1007/s00011-023-01692-5

**Published:** 2023-01-19

**Authors:** Abhinav Singla, Sebastian Reuter, Christian Taube, Marcus Peters, Karin Peters

**Affiliations:** 1 Department of Pulmonary Medicine, University Medical Center Essen-Ruhrlandklinik, Essen, Germany; 2 Department of Molecular Immunology, Ruhr-University Bochum, Universitätsstraße 150, 44801 Bochum, Germany

**Keywords:** Wingless-Type MMTV Integration Site Family, Member 5A, Airway remodeling, Asthma, Idiopathic pulmonary fibrosis, Chronic obstructive pulmonary disease

## Abstract

**Introduction:**

Chronic inflammatory lung diseases are a common cause of suffering and death. Chronic obstructive pulmonary disease (COPD) is the reason for 6% of all deaths worldwide. A total of 262 million people are affected by asthma and 461,000 people died in 2019. Idiopathic pulmonary fibrosis (IPF) is diagnosed in 3 million people worldwide, with an onset over the age of 50 with a mean survival of only 24–30 months﻿. These three diseases have in common that remodeling of the lung tissue takes place, which is responsible for an irreversible decline of lung function. Pathological lung remodeling is mediated by a complex interaction of different, often misguided, repair processes regulated by a variety of mediators. One group of these, as has recently become known, are the Wnt ligands. In addition to their well-characterized role in embryogenesis, this group of glycoproteins is also involved in immunological and structural repair processes. Depending on the combination of the Wnt ligand with its receptors and co-receptors, canonical and noncanonical signaling cascades can be induced. Wnt5A is a mediator that is described mainly in noncanonical Wnt signaling and has been shown to play an important role in different inflammatory diseases and malignancies.

**Objectives:**

In this review, we summarize the literature available regarding the role of Wnt5A as an immune modulator and its role in the development of asthma, COPD and IPF. We will focus specifically on what is known about Wnt5A concerning its role in the remodeling processes involved in the chronification of the diseases.

**Conclusion:**

Wnt5A has been shown to be involved in all three inflammatory lung diseases. Since the ligand affects both structural and immunological processes, it is an interesting target for the treatment of lung diseases whose pathology involves a restructuring of the lung tissue triggered in part by an inflammatory immune response.

## Remodeling of lung tissue in chronic airway diseases

The prevalence of bronchial asthma in 0–17 years old in Germany is 6% and the third most common chronic disease [2]. A total of 262 million people are affected by asthma worldwide (WHO). The most common type of asthma is atopic asthma caused by an allergic inflammation in response to allergens, which leads to tissue damage. Tissue damage is induced by the major basic protein of eosinophils [3], by proteases of mast cells [4], or, in the case of inflammation associated with neutrophils, by neutrophil extracellular traps [5]. If the inflammatory reaction is of short duration, the damage can be completely eliminated by repair processes. Excessive repair processes occur in persistent inflammation, leading to the remodeling of the lung tissue, including hyperplasia of mucus-producing goblet cells, thickening of the basal lamina of the airway epithelium, neovascularization, and the formation of fibrotic foci below the basement membrane [6]. Taken together, these processes are responsible for an obstruction of the airways (mechanisms are summed up in detail by [7]).

Idiopathic fibrosis is the most lethal interstitial pneumonia of unknown cause. It is characterized by excessive fibrosis in the lung tissue with a massive production and deposition of extracellular matrix (ECM). The prevalence of idiopathic pulmonary fibrosis (IPF) in Germany is estimated to be about 2–29 out of every 100,000 people [1]. This disease has a poor outcome as the mean survival time is only 24–30 months. In contrast, to COPD and asthma inflammation seems to be not the starting point for the pathogenesis of IPF. The generation of fibrotic foci seems to precede the inflammatory response during the development of disease [8]. The foci seem to evolve in response to a repetitive injury of alveolar epithelial cells. The cause of the injury may be chronic inhalation of cigarette smoke or occupational contact to toxic chemicals or gases. Pharmaceutics such as cytostatic cancer medication may also initiate the disease, e.g., in a murine model, an IPF like disease can be induced by a single dosage of bleomycin. The deposition of collagens in the fibrotic foci results in a stiffening of the lung parenchyma resulting in restrictive lung disease.

Chronic obstructive pulmonary disease (COPD) is currently the fourth leading cause of death worldwide (with an upward trend) and is mainly caused by cigarette smoking. COPD is characterized by a reduction in lung function which is caused by the remodeling of the small airways and chronic bronchitis. Furthermore, according to the latest findings, it is a multifactorial systemic disease and is associated with cardiovascular diseases and other comorbidities. Chronic airway and systemic inflammation, mainly resulting from increased numbers and activation of neutrophils, alveolar macrophages, monocytes, and T-lymphocytes, are crucial for disease progression [9]. Emphysema is an additional feature of the remodeling in COPD, which is characterized by an abnormal and permanent enlargement of air spaces and the destruction of the lung parenchyma distal to the terminal bronchioles [10].

One pathological feature the three diseases have in common is fibrosis. However, the location of the fibrosis may differ. Where in asthma and COPD fibrotic tissue is predominantly found in the wall of the airway, in IPF fibrosis occurs in the interstitium.

### The central role of myofibroblasts in fibrosis of lung tissue

IPF, COPD, and asthma have in common that fibrotic processes are involved. The composition of the basement membrane proteins in asthma and COPD is abnormal and contributes to the severity of the disease [11]. While fibrosis in asthma is focused on the airway wall, in IPF, it occurs in the interstitium and is often characterized by the development of fibrotic foci. Hallmarks of these are the presence of myofibroblasts producing large amounts of ECM. TGF-β-1 (TGF β), which is released by both mast cells and granulocytes and promotes the formation of myofibroblasts, plays a key role in fibrotic disease [12]. The origin of these myofibroblasts can be diverse. Both systemic sources, such as fibrocytes recruited from bone marrow, and local sources, such as epithelial–mesenchymal transition or the conversion of resident fibroblasts and smooth muscle cells (SMCs), can play a role in the development of myofibroblasts. The cytokine TGFβ plays a decisive role in epithelial–mesenchymal transition [13]. Moreover, TGFβ-independent pathways induced by interleukin (IL)-4 and IL-13 are known to initiate the conversion of resident fibroblasts to myofibroblasts [14]. In addition to this function, IL-4 has also been shown to activate collagen production, thereby further promoting airway remodeling and fibrosis in heart muscle tissue [15]. The SMC change from a contractile to a synthetic phenotype with an increase and compositional change of ECM [16, 17]. Not only an increased production of ECM but also the capability of secreting chemokines give the cell an important role in the pathogenesis of asthma. Several studies have shown that the SMCs secrete chemokines, including eotaxin, CXCL10, and CX3CL1, which attract eosinophils and mast cells to the lung [18–20].

**Fig. 1:**
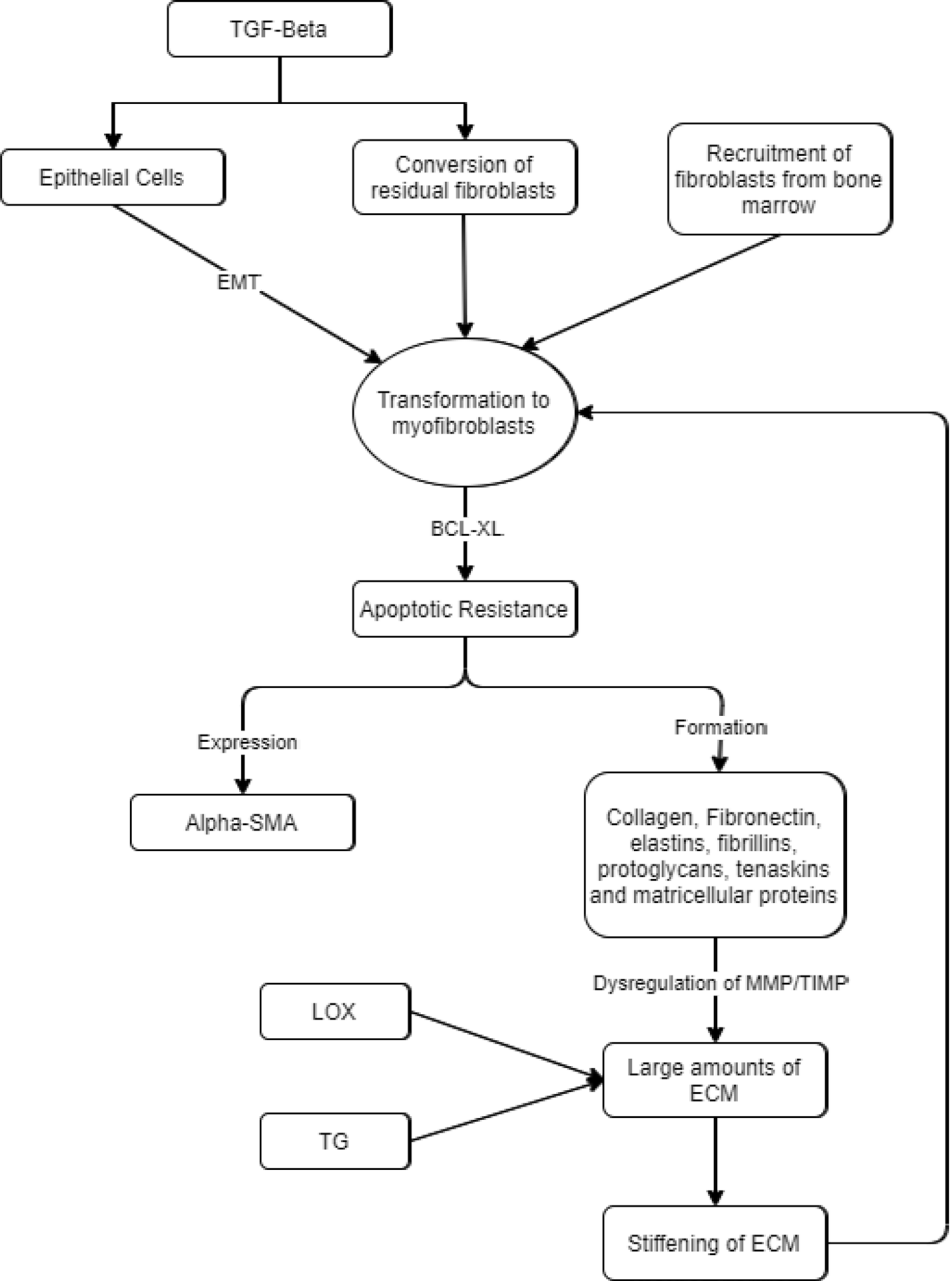
Role of fibroblasts in tissue remodeling (*TGF-Beta* transforming growth factor-Beta, *Alpha-SMA* alpha smooth muscle cells, *MMP* matrix metalloproteinase, *TIMP* tissue inhibitor of metalloproteinase, *ECM* extracellular matrix, *LOX* lysyl oxidase-like protein, *TG* transglutaminase)

In addition to structural cells, immune cells, such as macrophages, also play a role in the development of fibrosis. Hou et al. [21] showed that M2 macrophages in IPF promote the formation of myofibroblasts from resident mesenchymal stem cells in the lung. Both hyperplasia and hypertrophy are observed, which leads to the narrowing of the airways. Under physiological conditions, myofibroblasts are involved in the wound-healing process. Since they are contractile due to the expression of alpha smooth muscle actin (αSMA), they contract the wound edges. In addition, they produce collagens, fibronectins, elastins, fibrillins, proteoglycans, tenascins, and matricellular proteins to repair the injured tissue [22]. As soon as healing is completed, the myofibroblasts disappear by apoptosis [23]. On the contrary, the myofibroblasts are not eliminated by apoptosis in irreversible airway remodeling [24, 25] (Fig. 1). The myofibroblasts in fibrotic tissue continue to produce large amounts of ECM which is not degraded and leads to a stiffening of the tissue. Huang [26] shows that the stiffening of the ECM causes apoptosis resistance of the myofibroblasts. This process is mediated through BCL-XL. The latter is a transmembrane protein in mitochondria that has an anti-apoptotic effect by preventing permeabilization of the outer mitochondrial membrane. BH3 mimetic ABT-263 can overcome this anti-apoptotic effect, which induces apoptosis. A further cause of the stiffening of the ECM is possibly the dysregulation of matrix metalloproteinases and their natural tissue inhibitors [16, 27, 28]. The function of matrix metalloproteinases is the degradation of ECM. Furthermore, the ECM is cross-linked by transglutaminases, which further complicates its degradation [22]. Another family of enzymes involved in cross-linking are the so-called lysyl oxidase-like enzymes 1–4. The cross-linking of the ECM by these enzymes impedes its degradation, which leads to a further stiffening of the tissue. Lysyl oxidase-like enzyme 2 initiates the cross-linking of collagen and elastin. This increased cross-linking causes the activation of fibroblasts. It has already been shown that the inhibition of lysyl oxidase-like enzyme 2 in the murine bleomycin model is effective against pulmonary fibrosis [29]. The stiffening of a tissue generally leads to the conversion of fibroblasts to myofibroblasts, which, in turn, leads to the development of fibrosis in the lung tissues.

### The receptors of Wnt5A and their manifold ways of signal transduction

The ligand Wnt5A belongs to the family of wingless-type MMTV integration site proteins. This family consists of secreted lipid-modified glycoproteins that bind to receptors of the Frizzled family (Fz/Fzd) and to the receptors tyrosine kinase-like orphan receptor 2 (ROR2) and Ryk. To date, 19 different WNT factors and 10 different Frizzled receptors have been identified in mouse and human. In addition, the core receptors lipoprotein receptor-related proteins 5 and 6 are involved as co-receptors in Wnt-mediated signaling. The combination of Wnt/Fzd binding, the involvement and type of co-receptors and the cellular context determine the intracellular signal transduction. The three most important signaling pathways are the canonical, beta-catenin-dependent, and two noncanonical pathways, namely, the calcium-dependent and the planar cell polarity signaling pathway. Numerous publications show that Wnt5A is a prototype ligand for noncanonical Wnt signaling, which, with a few exceptions [30], induces beta-catenin-independent signaling cascades.

The Wnt5A signaling plays an important role in developmental biological processes, which is proved by a massive impairment of the embryonic development of the mouse lung after the ubiquitous elimination of Wnt5A. It results in an increase in peripheral branching, which leads to an increased number of terminal airways. In addition, lung maturation is inhibited, as reflected by the persistence of thickened intersaccular interstitium [31]. By contrast, overexpression of Wnt5A in the lung causes reduced branching, dilated airways, and abnormal lobation [32]. These results from mouse models prove the essentiality of Wnt5A in correct epithelial–mesenchymal interaction in the lung. Furthermore, it could be shown that the lungs of Wnt5A and ROR2 knock-out mice show a very similar phenotype [33]. Such an interaction of Wnt5A and ROR2 in epithelial branching could also be shown in breast tissue [34]. Another important receptor for Wnt5A is the receptor tyrosine kinase RYK which regulates the planar cell polarity signaling pathway [35]. The role of RYK in fibrosis processes in the lung is not yet known. Other known receptors for Wnt5A are the Frizzled receptors 2, 3, 4, 5, 6, 7, 8 and CD146 [36]. Fzd 2 is known to be important for the branching of the epithelium during lung development, because it influences the shape of the epithelial cells [32]. Wnt5A produced by macrophages can also induce angiogenesis and lymphangiogenesis in the lung [37]. There are two different isoforms of Wnt5A: Wnt5A-S and Wnt5A-L, where the expression of Wnt5A-S is controlled by an alternative promoter. Wnt5A-S contains 19 amino acids fewer than Wnt5A-L at the N-terminus, but otherwise has an identical structure [38, 39]. There are indications that the isoforms differ in their function. While Wnt5A-L inhibits proliferation, Wnt5A-S is pro-proliferative in tumor cells of different origin. Huang et al. [40] show that Wnt5A-S is more strongly expressed in colorectal cancer than Wnt5A-L. Wnt5A-S in the colon carcinoma cell line HCT116 was down-regulated by siRNA, cell division was inhibited, and apoptosis was enhanced by an increased expression of FASLG. Furthermore, Huang et al. found a positive correlation between a high expression of Wnt5A-S and beta-catenin in combination with a low expression of Wnt5A-L. However, the two isoforms have not been considered separately in studies of pulmonary fibrosis, although their functions seem to be different. Two sources of Wnt5A are frequently used in experiments: one is recombinant WNT5A from Bio-Techne (formerly R&D), which corresponds to Wnt5A-L, and the cell line CRL-2814 from ATCC, which overexpresses Wnt5A-S stably [30]. Mikels was able to show that Wnt5A-S can activate the canonical pathway when FZD4 and the co-receptor LDL receptor-related protein 5 (LRP5) was present, but as soon as ROR2 was also expressed, Wnt5A-S inhibited the canonical pathway regardless of the calcium concentration in the cell.

### Function of Wnt5A as an immune modulator

There has been growing evidence in recent years that Wnt signaling pathways are also, in addition to their well-described role in embryogenesis and tissue homeostasis, involved in immune regulatory processes [36]. In vivo models in animals and murine and human in vitro models lead us to assume that canonical and noncanonical Wnt ligands can modulate cells of the innate and adaptive immunity and are involved in disease-modifying processes. Here, not only pro-inflammatory disease-driving but also the anti-inflammatory beneficial effects of Wnt ligands could be observed, depending on the model, cell type, disease, and Wnt ligand.

Numerous favorable immune-suppressing effects have been described in vitro and in several inflammation-dependent in vivo disease models for the modulation of the canonical β catenin-dependent pathway by Wnt ligands or antagonists. By comparison, immune regulatory data concerning the noncanonical Wnt ligands, especially Wnt5A, are sparse. Hereafter, recently published anti- and pro-inflammatory effects of the ligand will be reviewed.

Innate immune cells, such as macrophages and granulocytes, represent the first defensive lines against bacterial and viral infections. Both bacterial and viral pathogens can modulate the expression of canonical and noncanonical Wnt molecules upon infection in target tissues and specific cell types. The strength, type, and direction of the manipulation depend strongly on the pathogen, organ, or cell type [41]. Even though the number of pieces increases, there are still a lot of white spots in the puzzles which prevent us clearly seeing the complete picture of the pathogen Wnt interaction and the consequences for host and intruder. The complexity of Wnt signaling due to the large number of ligands, receptors, and co-receptors and their interaction makes it hard to decipher the functional impact of the pathway on the cellular and organ level in the context of infection and immune regulation. Nevertheless, there several studies which clearly demonstrate that Wnt ligands can affect central anti-pathogenic processes in macrophages and dendritic cells (DCs) and, thus, support or suppress the effectiveness of these immune cells against pathogens. Both canonical and noncanonical ligands have demonstrated promoting, inhibitory and neutral effects on processes such as phagocytosis, autophagy, and the production of oxygen species or inflammatory/anti-inflammatory cytokines. Wnt5A positively effects phagocytosis in RAW 264.7 macrophages [42].

Moreover, it is associated with the induction of pro-inflammatory cytokines not only in macrophages [43, 44] but also in other cell lines, such as endothelial cells, fibroblasts [45], human dental pulp cells [46], or bone marrow stromal cells [47], while its blockade increases anti-inflammatory IL-10 [42]. As summarized by [37], Wnt5A produced by macrophages acts in an autocrine manner by maintaining the immune functions of macrophages, and stimulates the release of inflammatory cytokines that are in a positive feedback loop with Wnt5A. To further illustrate the complexity of Wnt5A signaling, it is worth mentioning that tolerogenic Wnt5A properties in context with macrophages are also described, for example, in sepsis and breast cancer patients [48]. In addition to macrophages, Wnt ligands also have an immune modulatory impact on DCs, which play a central role in the modulation of adaptive immune responses and are associated with the pathogenesis of a variety of diseases [49].

Due to their ability to regulate the fate of adaptive immune responses and, thus, decide on tolerance or immunity, DCs have a key position in the development and progression of lung diseases such as asthma [50], [51].

At the same time, these properties also make them an interesting target for therapeutic intervention strategies. Canonical and noncanonical Wnt5A ligands are described to program a tolerogenic phenotype in DCs [52]. During the differentiation of DCs, Wnt5A lowers the DCs’ capacity for the uptake of antigens and leads to an increased production of IL-10 after Toll-like receptor stimulation. Changes in the energy metabolism and induction of IDO seem to be responsible for the tolerization of DCs, which are also capable to support the development of regulatory T cells [53].

These data suggest that Wnt5a may have an immunosuppressing function which could be beneficial in the context of allergies, such as allergic asthma. This will be further discussed in the next chapter. On the other hand, these immunosuppressive properties could have a negative impact on cancerous diseases, where effective immune responses are desirable.

Interestingly, in addition to the two antigen-presenting cells, granulocytes are also affected by Wnt ligands. Wnt5A induces the chemotaxis of neutrophils in vitro [54] and failures of Wnt signaling are associated with an increased infiltration of neutrophils into the skin in a murine psoriasis model [55].

Taken together, both the canonical and the noncanonical pathway can modulate the function of cells of the innate immunity. Here, pro- and anti-inflammatory effects can be observed depending on the cell type, microenvironment, and Wnt ligands. Noncanonical Wnt5A signaling is not only often associated with inflammation, but tolerogenic properties are also described, especially on DCs.

In the following, we will take a detailed look at the role of Wnt5A in the remodeling and modulation of inflammatory and immunological processes in different lung diseases.

### The role of Wnt5A in inflammation and remodeling in asthma, COPD and IPF

Regardless of the different isoforms, numerous results suggest that Wnt signaling plays a role in several chronic respiratory diseases [56]. What is known about Wnt5A in allergic asthma, IPF and COPD, and its role in the pathogenesis of these diseases?

#### Asthma

Syed et al. [57] found an enhanced transcription of the Wnt5A gene by incubating peripheral blood mononuclear cells from healthy donors with the asthma-associated cytokines IL-13 or IL-4. These results suggest that Wnt5A may be also released in the lung of patients suffering from Th2-associated allergic asthma, since it is known that IL-4 and IL-13 are abundantly present in the airways of those patients. The produced Wnt5A may affect locally immune cells, for instance mast cells, which are associated with asthma [58] and other allergic diseases. Wnt5A seems to be involved in maturation of mast cells [59]. Mast cells are well-known players in the Th2 endotype of asthma and it is known that these cells become activated by allergen-induced cross-linking of IgE on their surface. The activation of mast cells leads to the release of many bioactive molecules including several proteolytic enzymes which can have detrimental functions [60]. As discussed above, proteases are also released after Wnt5A stimulation of mast cells what may contribute to the detrimental activity of these cells in the absence of allergens.

Beside immune cells, structural cells are involved in asthma and some of them are known to release Wnt5A. For instance, eosinophilic granulocytes can stimulate the SMCs of the respiratory tract to produce TGFβ and Wnt5A [61]. This is in line with the fact that Wnt5A is also upregulated in airway SMCs in asthmatics [62]. The authors inhibited Wnt5A in SMCs with siRNA and saw that the TGFβ-dependent production of alpha-1 type 1 collagen and fibronectin is reduced. Hyperreactivity of SMCs is a hallmark of asthma and the basis for reversible obstruction of the airways. In this context, Wnt5A can increase the isometric contraction of tracheal SMCs in a calcium-independent manner [63]. Furthermore, Wnt5A drives actin cytoskeletal reorganization, but is not sufficient to increase the abundance of αSMA. These results may indicate that Wnt5A in asthma is also involved in airway obstruction.

**Fig. 2:**
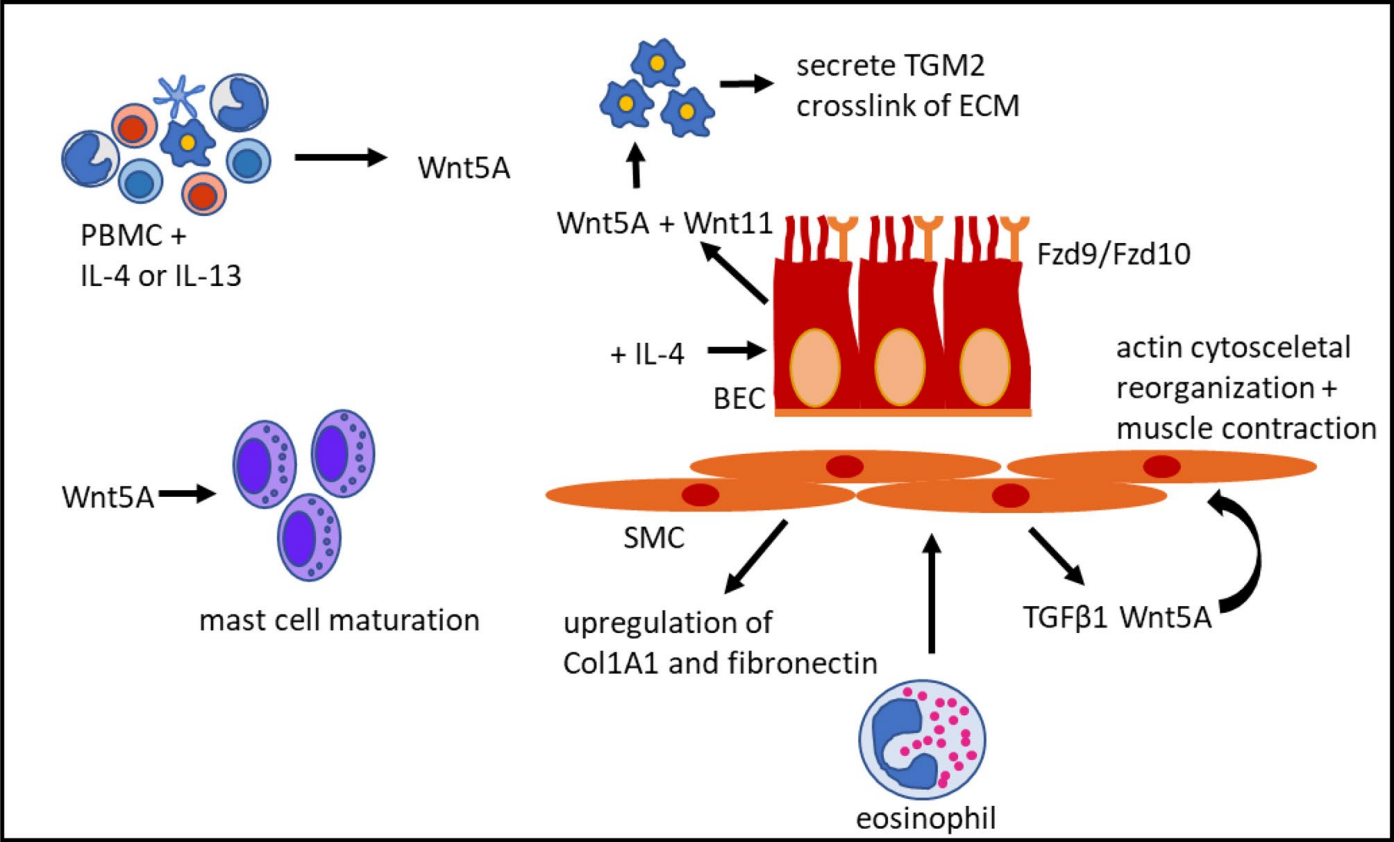
Wnt5A in asthma. Visual summary of the major points described in the text. *PBMC* peripheral blood mononuclear cells, *BEC* bronchial epithelial cells, *SMC* airway smooth muscle cells, *ECM* extracellular matrix, *TGM2* transglutaminase 2 and *Col1A1* alpha-1 type 1 collagen

In addition to SMCs, epithelial cells from human and mice are a source of Wnt5A. Dietz et al. [64] were able to show that normal human bronchial epithelial cells were stimulated to increase the expression of Wnt5A and Wnt11 as well as the Fzd receptors 9 and 10 by the addition of IL-4 (Fig. 2). An age-dependent increase of Wnt5A was shown in a house dust mite-dependent mouse asthma model, which, in turn, causes an increase in transglutaminase 2. In detail, the Wnt5A produced by bronchial epithelial cells induces the expression of transglutaminase 2 in macrophages (Fig. 2**)**. This, in turn, can lead to an increase in fibrosis, as it has the property of cross-linking matrix proteins and, thus, making them inaccessible for degradation [65].

Besides the action of Wnt5A on mast cells, epithelial cells, macrophages, and SMCs, it was shown that Wnt5A acts on fibroblasts. Specifically, a synergism of Wnt5A and IL-17A with respect to TGFβ secretion was shown after stimulation of primary murine fibroblasts from the lung [66]. Since TGFβ is an important driver of remodeling, this observation is of particular importance. Koopman et al. analyzed the cell type-specific contribution of the noncanonical Wnt ligand in allergic asthma by choosing a murine tetracycline-inducible SMC-specific Wnt5A overexpression model [67]. Interestingly, the increased expression of Wnt5A during allergen challenge enhanced airway remodeling processes such as the production of mucus and a trend toward increased smooth muscle layers around the airways but also seems to positively affect the inflammatory Th2 response in animals.

Many of these observations point to a role of Wnt5A as a bad guy in asthma. However, Reuter et al. demonstrated that Wnt ligands may also have a beneficial effect by limiting the Th2 response. They showed that doxycycline-induced lung-specific expression of canonical Wnt1 ligands and pharmaceutical intervention with recombinant Wnt1 ligands attenuates the asthma phenotype in animals [68], [69]. Interestingly, DCs seem to be especially responsible for the effects observed. In their work, they observed a Wnt1-dependent reduced migration of allergen-loaded DCs in vivo and an attenuated allergen-specific interaction between DCs and T cells in vitro. Excitingly, noncanonical Wnt5A was also able to suppress DC T-cell functions in vitro but failed to demonstrate a Wnt1 comparable therapeutic efficacy in vivo [69].

Interestingly, it was discovered in a genome-wide association study that Wnt5A is a novel locus for asthma exacerbations despite treatment with inhaled corticosteroids in European but not in non-European populations [70]. Hachim et al. [71] showed that Wnt5A and four other members of noncanonical Wnt signaling are down-regulated in the bronchial epithelium of patients suffering from severe asthma.

Altogether these results imply that the role of Wnt5A in asthma may depend on the timing of its release. If it is released early during the sensitization phase, it may protect from activation of the Th2 response by interfering the interaction of DCs and T helper cells, thus, preventing sensitization. However, if it is released, when the disease is already established, then it may lead to exacerbation by triggering the inflammatory immune response and promoting remodeling of lung tissue.

### COPD

**Fig. 3:**
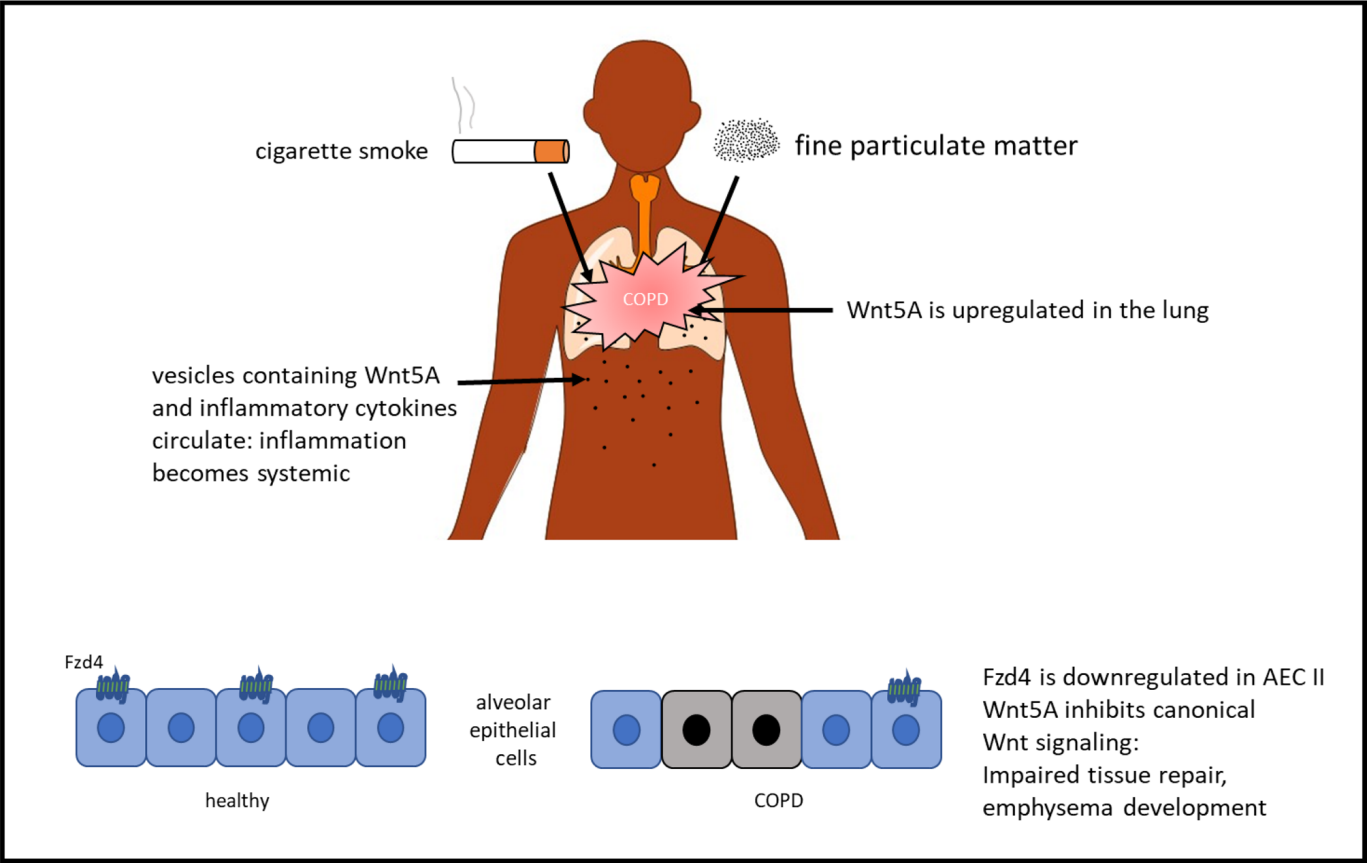
Role of Wnt5A in COPD. *Fzd4* Frizzled 4, *AEC* alveolar epithelial cells

Overall, there are less data for the involvement of Wnt5A in disease progression for COPD than for asthma. Baarsma et al. [72] showed that Wnt5A is upregulated in COPD in a mouse model of chronic smoke exposure and also in patients suffering from COPD. Upregulation was shown on the level of RNA transcription and also in Wnt5A protein expression in the lung tissue. Furthermore, it was shown that fibroblast-derived Wnt5A impairs wound healing. This observation was explained by the Wnt5A-mediated attenuation of beta-catenin-dependent canonical signaling in alveolar epithelial cells and therefore may contribute to emphysema formation. Wnt5A-S was shown to act via Fzd4 activating canonical Wnt signal transduction [30]. Skronska-Wasek et al. showed that Fzd4 is down-regulated in ATII cells of COPD patients [73]. Additionally, cigarette smoke exposure down-regulated Fzd4 in ATII cells in in vivo and in vitro experiments (Fig. 3). Thus, on the one hand, Wnt5A may block the canonical signaling [74] and, on the other hand, cannot induce canonical signaling via Fzd4 together resulting in reduced repair of the alveolar epithelium.

Feller et al. [75] observed that cigarette smoke-induced pulmonary inflammation becomes systemic by circulating vesicles. These vesicles contain Wnt5A and inflammatory cytokines and can distribute all over the body as they are found in the blood of COPD patients. Fine particulate matter ≤ 2.5 µm (PM 2.5) aggravates cigarette smoke-induced inflammation via the Wnt5A–ERK pathway in COPD [76]. Particulate matter 2.5 is sufficient to induce COPD in animal models. It induces the expression of inflammatory cytokines via the Wnt5A/Ror2 pathway in human bronchial epithelial cells. This was shown by performing siRNA experiments [77]. Obviously, Wnt5A is upregulated in COPD in several cell types of the lung and worsens inflammation and remodeling.

Taken together, these results indicate a role of Wnt5A in COPD in triggering and worsening the inflammatory response during the course of the disease. Moreover, Wnt5A interferes with the repair of the alveolar epithelium. Thus, it would be interesting to know whether Wnt5A also contributes to reduced repair of alveolar epithelium in IPF.

### IPF

**Fig. 4:**
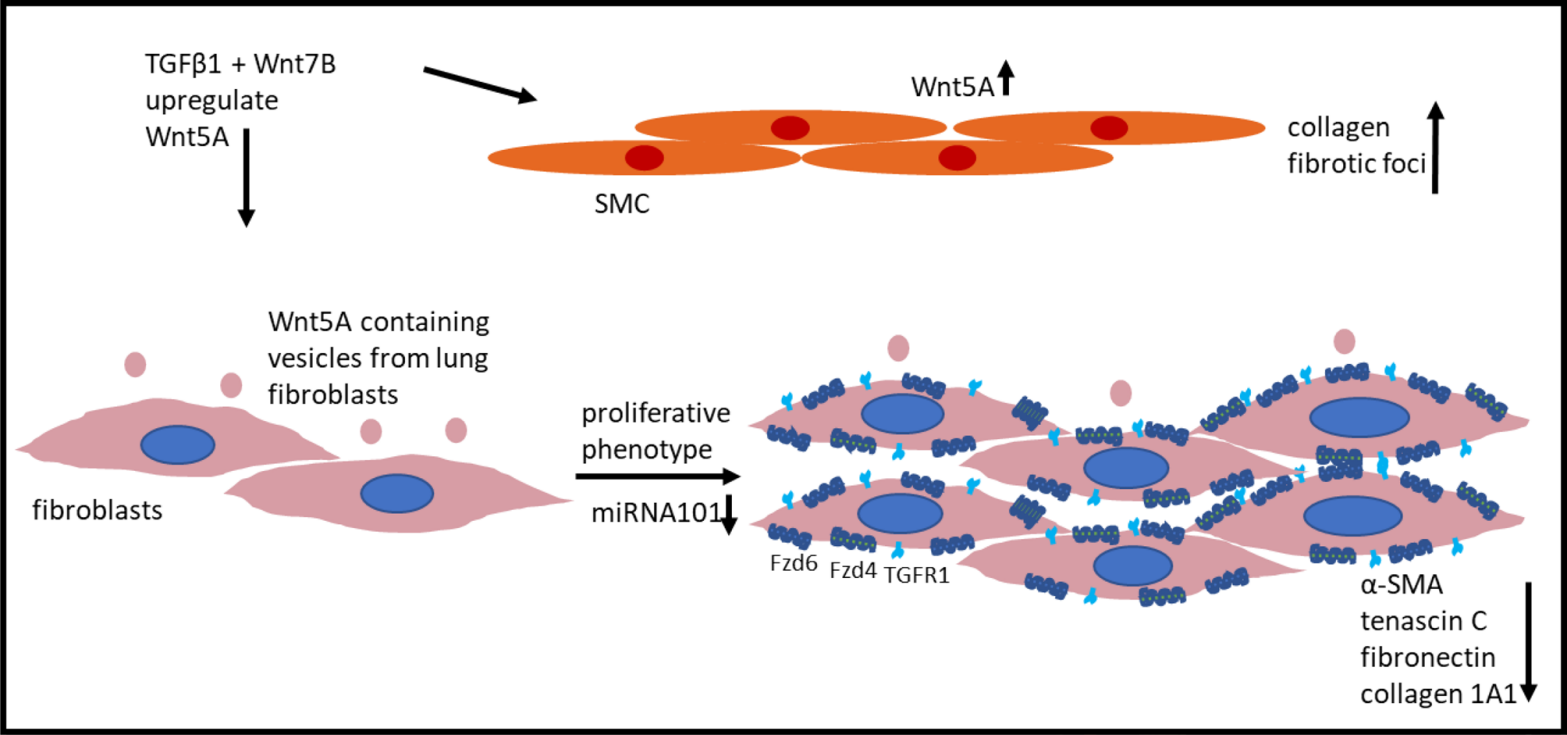
Wnt5A contributes to IPF. *SMC* smooth muscle cells

The Wnt5A expression in IPF is found in the airway and alveolar epithelium, SMCs, endothelium, airway epithelium, fibroblasts, and myofibroblasts in fibrotic foci [78]. This was shown by detection of the protein by immunohistochemically staining. Moreover, the authors showed that Wnt5A production is induced by Wnt7B and TGFβ1. Martin-Medina showed that extracellular vesicles containing Wnt5A are present in the bronchioalveolar lavage of IPF patients, leading to increased proliferation through the mediation of Wnt5A on pulmonary fibroblasts [79]. However, an induction of myofibroblasts could not be recognized in this study. This was proved by the downregulation of myofibroblast markers, such as αSMA, fibronectin, collagen 1A1 and tenascin C, by Wnt5A (Fig. 4). By contrast, Vuga et al. [24] showed that Wnt5A stimulates fibroblasts to secrete more fibronectin. This contradiction may originate from the different isoforms of Wnt5A that were not differentiated in all of these studies. Huang et al. [80] observed that MicroRNA 101 is down-regulated in the lungs of IPF patients (Fig. 4). They showed that forced overexpression of this mi-RNA attenuates fibrosis. The authors ruled out that it suppresses Wnt5A-driven fibroblast proliferation by inhibiting NFATc2 signaling via targeting FZD4/6 expression and TGFβ1-induced activation of fibroblasts. It was recently shown by Carmo-Fernandes et al. [81] that a smooth muscle-restricted Wnt5A knock-out in bleomycin-induced lung fibrosis reduced the deposition of collagen and the number of fibrotic foci in the lung. Lung function conditions were improved and weight loss reduced compared to wild-type mice. These data prove a profibrotic role of Wnt5A in lung fibrosis.

Altogether these data show that Wnt5A in IPF plays a profibrotic role triggering the release of collagen and establishing fibrotic foci in lung parenchyma. However, it is interesting to speculate that inflammation in the IPF lung may also increase, similar to COPD, and conversely, fibrosis in COPD may be increased by the action of Wnt5A. Yet, it is important that findings regarding the Wnt5A-driven pathogenesis under one disease condition should be studied for the other conditions to highlight similarities and differences in the compared diseases.

## Conclusion

All in all, the role of Wnt5A in lung diseases is still inconsistent. Due to the organ–cell- and context-dependent expression of Wnt5A and its receptors, its effect and role in the lung and the organ-specific diseases is not easy to determine. Nevertheless, since the ligand affects both structural and immunological processes, it is an interesting target to treat lung diseases whose pathology often involves both structural and immunological components. New methods, such as the use of genetically modified mice, which allow a targeted manipulation of Wnt5A expression help us to understand its function in the lungs and associated diseases better. This targeted manipulation not only allows us to distinguish effects associated with embryogenesis from its function in homeostasis and inflammation, but also offers the possibility of investigating the cell-specific role of the ligand. Furthermore, new human in vitro models and their manipulation by recombinant ligands or manipulation of the expression by siRNA or CrispR/Cas methods also allow the investigation of the function of Wnt5A in humans.

These possibilities of a detailed elucidation of Wnt5A can offer new starting points for Wnt-based therapeutic approaches for lung diseases such as asthma, COPD, or IPF in the future. However, one must be aware of the difficulties accompanying clinical trials dealing with the manipulation of WNT-signaling. WNT factors are involved in several homeostatic processes; therefore, it is probable that manipulation may come at the cost of side effects. Thus, it is even more important to evaluate the precise role of the different WNT factors in health and disease to gather all the information possible before translating to clinical trials.

## Data Availability

Not applicable.
